# Identification of *VIPR2* rare and common variants in the Chinese Han population with schizophrenia

**DOI:** 10.3389/fnmol.2023.1170708

**Published:** 2023-04-27

**Authors:** Jiajun Yin, Juan Zhou, Fang Fang, Shui Yu, Jun Wang, Jianmin Yuan, Zhenhe Zhou

**Affiliations:** ^1^The Affiliated Wuxi Mental Health Center of Jiangnan University, Wuxi Central Rehabilitation Hospital, Wuxi, China; ^2^Shandong Cancer Hospital and Institute, Shandong First Medical University and Shandong Academy of Medical Sciences, Jinan, Shandong, China

**Keywords:** *VIPR2* gene, schizophrenia, amplicon targeted resequencing, association study, causative variants

## Abstract

**Introduction:**

Schizophrenia is a severe and chronic psychiatric disorder with hereditary risk up to 80% as previous studies indicated. Several researches have demonstrated a significant association between schizophrenia and microduplications that overlap the vasoactive intestinal peptide receptor 2 gene (*VIPR2*).

**Methods:**

To further investigate potential causal *VIPR2* gene variants, all exons and un-translated portions of the *VIPR2* gene were sequenced using amplicon targeted resequencing in 1804 Chinese Han patients with schizophrenia and 996 healthy counterparts in the present study.

**Results:**

Nineteen rare non-synonymous mutations and 1 frameshift deletion was identified for schizophrenia, among which 5 variants have never been reported so far. Frequencies of rare non-synonymous mutations were significantly different between the two groups. Specifically, the non-synonymous mutation rs78564798 (*P_allele_* = 0.006) as well as two rare variations in the *VIPR2* gene’s introns (rs372544903, *P_allele_* = 0.026 and a novel mutation, chr7:159034078, GRCh38, *P_allele_* = 0.048) were significantly associated with schizophrenia.

**Discussion:**

Our findings add new evidence that the functional and probable causative variants of *VIPR2* gene may play an important role in susceptibility to schizophrenia. Further studies on validations of *VIPR2*’s function in the etiology of schizophrenia are warranted.

## Introduction

Schizophrenia (SCZ) is a severe psychiatric disorder that typically manifests in the late teens and early twenties and affects almost 1% of the world population (Marder and Cannon, 2019). Hallucinations, delusions, disorganized talk, diminished motivation and expression, and cognitive deficits are the core symptoms of SCZ (Joyce and Roiser, 2007; Owen et al., 2016). More than 50% of patients with SCZ experience long-term intermittent psychiatric problems, and about 20% experience chronic symptoms or even disability (Barbato, 1998). SCZ is currently one of the top 10 causes of disability worldwide (Fleischhacker et al., 2014), and those who have it typically live 10–20 years less than the general population (Chesney et al., 2014). Although the etiology and pathogenesis of SCZ have not been completely understood, it is generally acknowledged that heritable and environmental factors affect. Previous twin and family studies suggest heritability accounting for 60% ~ 80% of the risk of SCZ (Sullivan et al., 2003).

Multiple researches on the genetics of SCZ have shown numerous single-nucleotide polymorphisms (SNPs; Ripke et al., 2014; Li et al., 2017; Trubetskoy et al., 2022), rare coding variations (Purcell et al., 2014), and rare copy number variations (CNVs; Li et al., 2016; Marshall et al., 2017) that are linked to the risk of developing SCZ. It was first reported in 2011 that microduplications that overlapped the *VIPR2* gene or its upstream were significantly associated with SCZ (Levinson et al., 2011; Vacic et al., 2011). Subsequent researches conducted on the Han Chinese population further confirmed the significant association between SCZ and the *VIPR2* CNV (Yuan et al., 2014; Li et al., 2016). The *VIPR2* gene was also found to be differently methylated between SCZ patients and controls by genome-wide DNA methylation study on post-mortem human brain tissue (Wockner et al., 2014). Additionally, several studies showed that the *VIPR2* gene is associated with other psychiatric disorders, such as higher frequencies of CNVs in autism spectrum disorder (Firouzabadi et al., 2017), hypomethylation at CpG sites in attention deficit and hyperactivity disorder (ADHD; Wilmot et al., 2016) and different frequencies of SNP (rs885861) between patients with mood disorders (MD) and controls (Soria et al., 2010).

**Figure 1:**
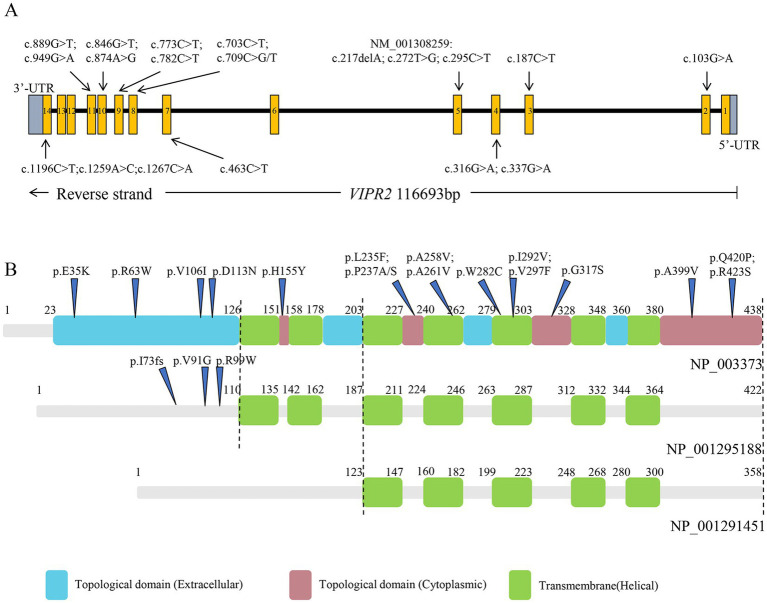
Structure of *VIPR2* gene and VPAC2 isoforms. **(A)** *VIPR2* gene structure, boxes 1 ~ 14 indicate the protein-coding exons. **(B)** VPAC2 isoforms structure based on NP_003373, NP_001295188, and NP_001291451.

The *VIPR2* gene is located on chromosome 7q36.3 and harbors 14 exons, which encodes a class B 7-transmembrane G-protein-coupled receptor (GPCR) called VPAC2. Three VPAC2 isoforms are depicted in Figure 1, including NP 003373 (438aa), NP 001295188 (422aa), and NP 001291451 (358aa). In humans, the VPAC2 receptor is widely present in multiple organs and brain. It is highly expressed in neurons of the cerebral cortex as well as the thalamus and hypothalamus, particularly in the suprachiasmatic nuclei (Sheward et al., 1995; An et al., 2012; Ago et al., 2021). Vasoactive intestinal peptide (VIP) and pituitary adenylate cyclase-activating polypeptide (PACAP) are two natural ligands that interact with the VPAC2 receptor which plays a crucial role in the physiology of the central nervous system (CNS) (Laburthe et al., 2007). As a GPCR, the VPAC2 receptor mainly triggers the activation of adenylate cyclase (AC), through coupling with Gs-type trimeric G-proteins, which subsequently produces cAMP and activates protein kinase A (PKA). Additionally, it has been discovered that the VPAC2 receptor also activates phospholipase C (PLC) through G proteins of Gq and Gi/Go families (MacKenzie et al., 2001; Laburthe et al., 2002, 2007).

Studies with VPAC2-overexpressing and VPAC2-deficient mice models have revealed that VPAC2 receptors are crucial for regulating circadian rhythms (Shen et al., 2000; Harmar et al., 2002) and have an effect on fear cognition (Ago et al., 2017). According to studies, VPAC2-deficient mice exhibited altered synaptic structure in their prefrontal cortex and selective deficits in fear extinction, which is a primary symptom of post-traumatic stress disorder (Milad et al., 2009; Ago et al., 2017). According to research on the spatiotemporal expression of VPAC2 in postpartum mice, the maturation of circuits that are involved in cognition may be disrupted by excessive, incorrectly timed or ectopic activation of VPAC2 receptors (Waschek et al., 1996; Ago et al., 2021). Recently, it is reported that the conditional human *VIPR2* CNV BAC transgenic mouse model of *VIPR2* CNV, known as hVIPR2-BAC tg mice, demonstrated cognitive, social behavior, and sensorimotor gating deficits as well as a decline in the complexity of the projection neurons (Tian et al., 2019; Ago et al., 2021). In cell culture models, as a response to the VPAC2 receptor and VIP agonists BAY 55–9,837, accumulation of *VIPR2* mRNA and cAMP was observed, highlighting the functional importance of the microduplications (Vacic et al., 2011).

Aforementioned evidence consistently indicates that the *VIPR2* gene is a risk gene for SCZ, and study results in cell culture and mice models suggest that VPAC2 receptor may impair cognitive function and sensory information processing by influencing neurological development, synaptic plasticity, and neuronal maturation, which may help explaining the potential role of *VIPR2* gene in the pathogenic mechanisms of SCZ. To further discover potential pathogenic variants of *VIPR2* gene for SCZ, amplicon targeted resequencing was performed for all exons and un-translated region (UTR) of *VIPR2* gene in 1804 SCZ patients and 996 healthy controls in the present study, and associations between SNPs, single nucleotide variants (SNVs), short insertions and deletions (InDels) of the *VIPR2* gene and SCZ were investigated.

## Materials and methods

### Subjects

A total of 1804 SCZ patients (693 women and 1,111 men; mean age = 44.51 years, SD = 12.13) were enrolled for this study and 996 healthy controls (559 women and 437 men; mean age = 43.41 years, SD = 20.17) without a history of mental health disorders or neurological diseases were recruited from local communities by advertisement (Table 1).

**Table 1:** Characteristics of the study sample set.

	*n*			Age, years	
	Men	Women	Total	Mean	s.d.
Patients with SCZ	1,111	693	1,804	44.51	12.13
Healthy controls	437	559	996	43.41	20.17

SCZ, schizophrenia; s.d., standard deviation.

All participants were independent Chinese Han from the Jiangsu Province of China and provided signed informed consent prior to participating in the study. All SCZ patients were recruited by the Wuxi Mental Health Center from 2015 to 2017 and diagnosed by at least two independent psychiatrists according to the DSM-IV criteria. Patients were excluded if they have suffered hypertension; diabetes; neurological illness; mood disorder; mental retardation; history of substance use and psychotic disorder due to general medical condition. The study was approved by the local Ethics Committees, and conducted according to the principles of the Declaration of Helsinki 1975.

### Amplicon targeted resequencing

Peripheral blood samples were collected from all participants using K_2_EDTA tubes, and DNA was extracted through LifeFeng Genomic DNA Purification Kit (Lifefeng Biotech Co., Ltd., Shanghai, China). DNA quality control was performed utilizing a NanoDrop 1,000 Spectrophotometer (Thermo Scientific, United States). Thirty-two pairs of primers divided into two pools were designed to cover the *VIPR2* gene. The primers’ sequences and their targeted regions are shown in Supplementary Table S1. Sequence libraries preparation was performed in a two-staged PCR process (Shanghai DynastyGene Co. Ltd). The size distribution of DNA library fragments was determined using the High Sensitivity DNA kit (Agilent Technologies, United States) on the Agilent 2,100 Bioanalyzer. Hundred and fifty bp paired-end reads were run on an Illumina HiSeq X ten platform (Illumina, United States).

### Variants identification and validation

Each data set was independently put through the pipeline of the Genome Analysis Toolkit (GATK) Best Practices^1^ for detecting germline short variants. Raw reads were aligned to the human reference genome (GRCh38) using the Burrows-Wheeler Aligner (BWA; Li and Durbin, 2010), SNPs and InDels were called using the GATK haplotypercaller, and the variations were annotated using Annovar (Wang et al., 2010). Alignments involving multiple species were carried out using the online Clustal Omega program.^2^ Rare non-synonymous mutations were confirmed by Sanger sequencing.

### Statistical analysis

**Figure 2:**
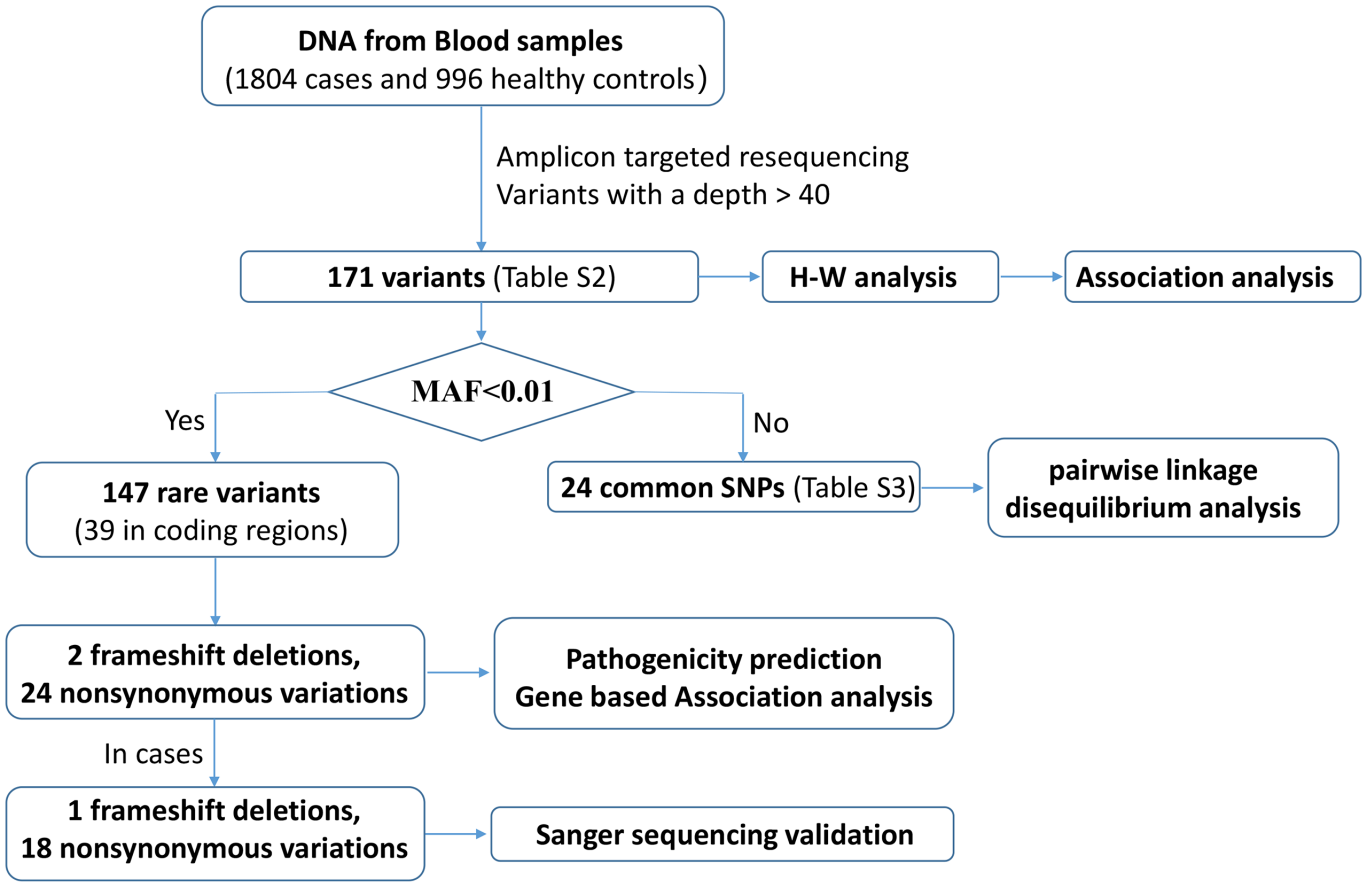
The pipeline of variants QC and analysis.

The SHEsisPlus online software platform^3^ (Yong and He, 2005; Li et al., 2009; Shen et al., 2016) was used to perform all single locus tests, including Hardy–Weinberg equilibrium (HWE) and association tests. Associations of allelic and genotypic polymorphisms with SCZ were analyzed using the chi-square test or Fisher’s exact test. All tests were two-tailed, and the *p*-values were calibrated using the false discovery rate (FDR-BH), which was regarded as statistical significance as being less than 0.05. Pairwise linkage disequilibrium (LD) analysis was performed for the common variants by Haploview software version 4.2^4^ and the algorithm of four gamete rule was used to define haplotype blocks. The QC and analysis pipeline are shown in Figure 2.

## Results

### Variants identification

We performed Sanger sequencing validation for 57 cases containing non-synonymous mutations or frameshift deletion detected by next-generation sequencing. Twenty variants harbored by 33 cases among them that all have next-generation sequencing depths deeper than 40, were confirmed to be true positives. The remaining 24 samples were all found to be false positives, 22 of which had depths lower than 40. Therefore, variants considered in the subsequent analysis were only those with a depth greater than 40.

A total of 173 high-quality variations, including 61 3’-UTR variants, 45 coding exon variants, 65 intron variants, one splicing area variant, and one downstream variant, were identified in the SCZ and control groups (Supplementary Table S2). In all, 147 loci with minor allele frequency (MAF) below 0.01 in across all databases, including the 1,000 Genomes Project, Exome Aggregation Consortium, and NHLBI Exome Sequencing Project, were chosen as rare variants. Of these, 70 loci were reported for the first time, and details of the other 24 common SNPs are provided in Supplementary Table S3.

### Analyses of rare variants in coding regions

**Figure 3:**
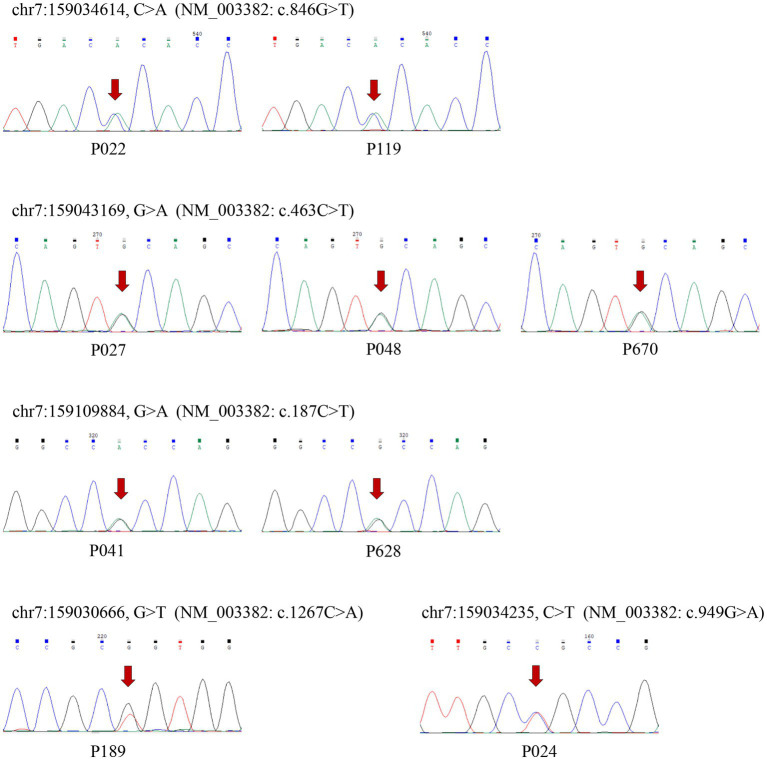
The results of Sanger sequence verifying the rare non-synonymous variants. The six variants were newly reported and arrows indicate the mutation sites.

Thirty-nine distinct variant loci, comprising two frameshift deletions, 13 synonymous mutations, and 25 non-synonymous variations that include two polymorphisms at the rs78564798 locus, were identified in the coding exons. Among whom, 5 synonymous mutations, 6 non-synonymous variants, and 2 frameshift deletions were initially reported. Figure 1 depicts the 19 non-synonymous variants and one frameshift deletion observed in cases. Sanger sequencing was utilized to confirm them and it was determined that all variants were heterozygous (Figure 3; Supplementary Figure S1).

The pathogenicity of the non-synonymous variants was predicted using SIFT, PolyPhen-2,^5^ MutationTaster,^6^ and LRT (Chun and Fay, 2009) in Annovar (Table 2). Only five non-synonymous variants (NM_003382: c.846G > T, c.463C > T, c.1267C > A, c.949G > A, c.187C > T) were predicted to be “D” (Deleterious for SIFT and LRT, Probably damaging for Polyphen-2, disease causing for MutationTaster) by at least three sorts of software. All of them were only detected in cases except rs534492909 (NM_003382: c.463C > T), and two of the five non-synonymous variants were firstly reported. Six variants which were only identified in control group were anticipated to be “D” by two or fewer pathogenicity prediction tools. The CADD (Combined Annotation Dependent Depletion) score was available at https://cadd.gs.washington.edu/snv for all variants. Scores of the five detrimental non-synonymous variants are all greater than 20, ranking them in the top 1% of deleterious variations in the human genome.

**Table 2:** Detailed information on rare functional variants detected in this study.

Position	Variants	Variants status	SIFT	Polyphen-2	MutationTaster	LRT	CADD	Novel or not	Individuals		MAF	
									case	control	ExAC_EAS	1000g2015aug_eas
chr7:159030666	NM_003382: c.1267C > A/p.R423S	Heterozygous	T	D	D	D	20.9	Novel	1	0	–	–
chr7:159034235	NM_003382: c.949G > A/p.G317S	Heterozygous	T	D	D	D	23.4	rs568217175	1	0	0.0003	–
chr7:159034614	NM_003382: c.846G > T/p.W282C	Heterozygous	D	D	D	D	29.5	Novel	2	0	–	–
chr7:159043169	NM_003382: c.463C > T/p.H155Y	Heterozygous	D	D	D	D	25.3	rs534492909	10	8	0.0045	0.002
chr7:159109884	NM_003382: c.187C > T/p.R63W	Heterozygous	D	D	D	N	27.8	rs750654613	2	0	0	–
chr7:159030674	NM_003382: c.1259A > C/p.Q420P	Heterozygous	D	B	D	N	22.2	rs184356169	1	1	0.0085	0.003
chr7:159030737	NM_003382: c.1196C > T/p.A399V	Heterozygous	T	B	N	N	15.29	Novel	1	0	–	–
chr7:159034295	NM_003382: c.889G > T/p.V297F	Heterozygous	T	B	N	N	22.5	rs759659222	1	0	0	–
chr7:159034586	NM_003382: c.874A > G/p.I292V	Heterozygous	T	P	D	D	19.26	rs199630455	3	8	0.001	0.002
chr7:159035979	NM_003382: c.782C > T/p.A261V	Heterozygous	T	B	N	N	0.179	rs150485248	13	10	0.0026	0.001
chr7:159035988	NM_003382: c.773C > T/p.A258V	Heterozygous	T	B	N	N	4.957	rs200955443	2	0	0.0013	0.001
chr7:159036791	NM_003382: c.709C > G/p.P237A	Heterozygous	T	B	N	N	19.81	rs78564798	1	10	0.0014	0.006
	NM_003382: c.709C > T/p.P237S	Heterozygous	T	B	N	N	17.74	rs78564798	1	0	0.0018	–
chr7:159036797	NM_003382: c.703C > T/p.L235F	Heterozygous	T	B	N	N	8.298	rs149197032	1	0	0	–
chr7:159096849	NM_001308259: c.295C > T/p.R99W	Heterozygous	D	.	N	.	0.161	rs530352260	5	0	0.0023	–
chr7:159096872	NM_001308259: c.272 T > G/p.V91G	Heterozygous	T	.	N	.	0.371	Novel	1	1	–	–
chr7:159096927	NM_001308259: c.217delA/p.I73fs	Heterozygous	.	.	.	.	.	Novel	2	0	–	–
chr7:159103777	NM_003382: c.337G > A/p.D113N	Heterozygous	T	B	N	D	12.7	rs149519347	1	0	0	–
chr7:159103798	NM_003382: c.316G > A/p.V106I	Heterozygous	T	B	N	N	0.402	rs143947210	1	0	0.0001	–
chr7:159142494	NM_003382: c.103G > A/p.E35K	Heterozygous	T	B	D	D	22.4	rs375596936	4	2	0.0014	–
chr7:159031846	NM_003382: c.1125delC/p.Y375fs	Heterozygous	.	.	.	.	.	Novel	0	1	–	–
chr7:159035992	NM_003382: c.769G > A/p.G257S	Heterozygous	T	B	N	N	10.58	rs749694333	0	1	0	–
chr7:159043066	NM_003382: c.566C > A/p.T189K	Heterozygous	T	P	N	N	22.3	rs770411003	0	2	0.0001	–
chr7:159096837	NM_001308259: c.307G > A/p.E103K	Heterozygous	T	.	N	.	0.079	Novel	0	1	–	–
chr7:159096852	NM_001308259: c.292C > T:p.R98W	Heterozygous	D	.	N	.	0.098	rs568908306	0	1	0	–
chr7:159109908	NM_003382: c.163G > A/p.V55I	Heterozygous	T	B	N	N	12.02	rs371596513	0	1	0.0001	–
chr7:159109919	NM_003382: c.152C > A/p.A51D	Heterozygous	D	P	D	N	26.7	Novel	0	2	–	–

D, Deleterious for SIFT_pred and LRT, Probably damaging for Polyphen-2_pred, disease_causing for MutationTaster_pred; T, tolerated; P, possibly damaging; B, benign; N, neutral. SCZ, schizophrenia; CADD, combined annotation dependent depletion.

**Figure 4:**
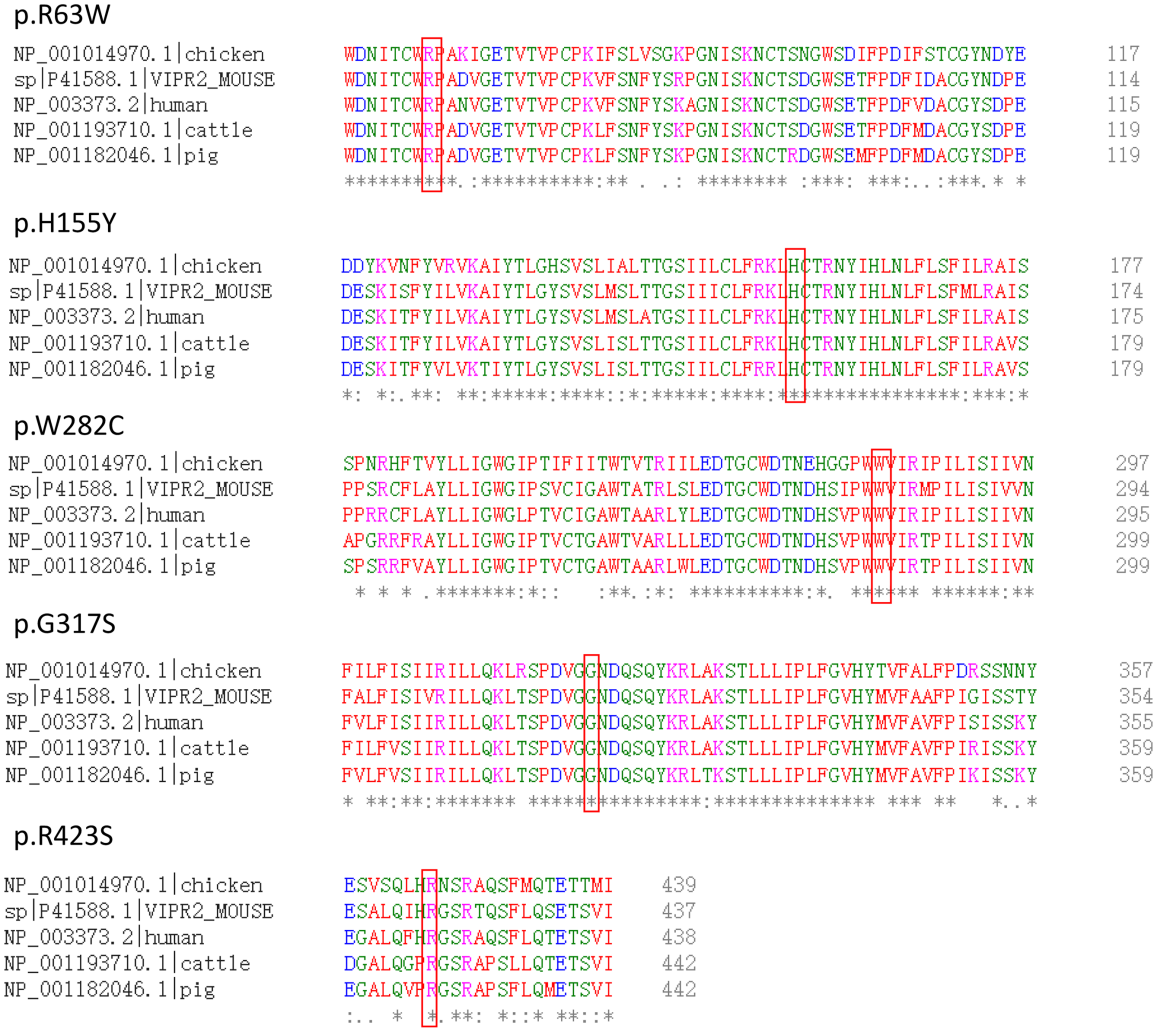
Multiple alignments of VPAC2 protein sequences of various species. The sites of non-synonymous variants were box out.

The amino acid variants for the five detrimental non-synonymous variants are *VIPR2* (NP_003373): p.(Trp282Cys), p.(His155Tyr), p.(Arg423Ser), p.(Gly317Ser), and p.(Arg63Trp), respectively. Multiple alignments of VPAC2 receptor sequences from human, mouse, pig, cattle, and chicken showed that all the five loci are mostly conserved across evolution (Figure 4).

### Association analysis of the variants with SCZ

**Figure 5:**
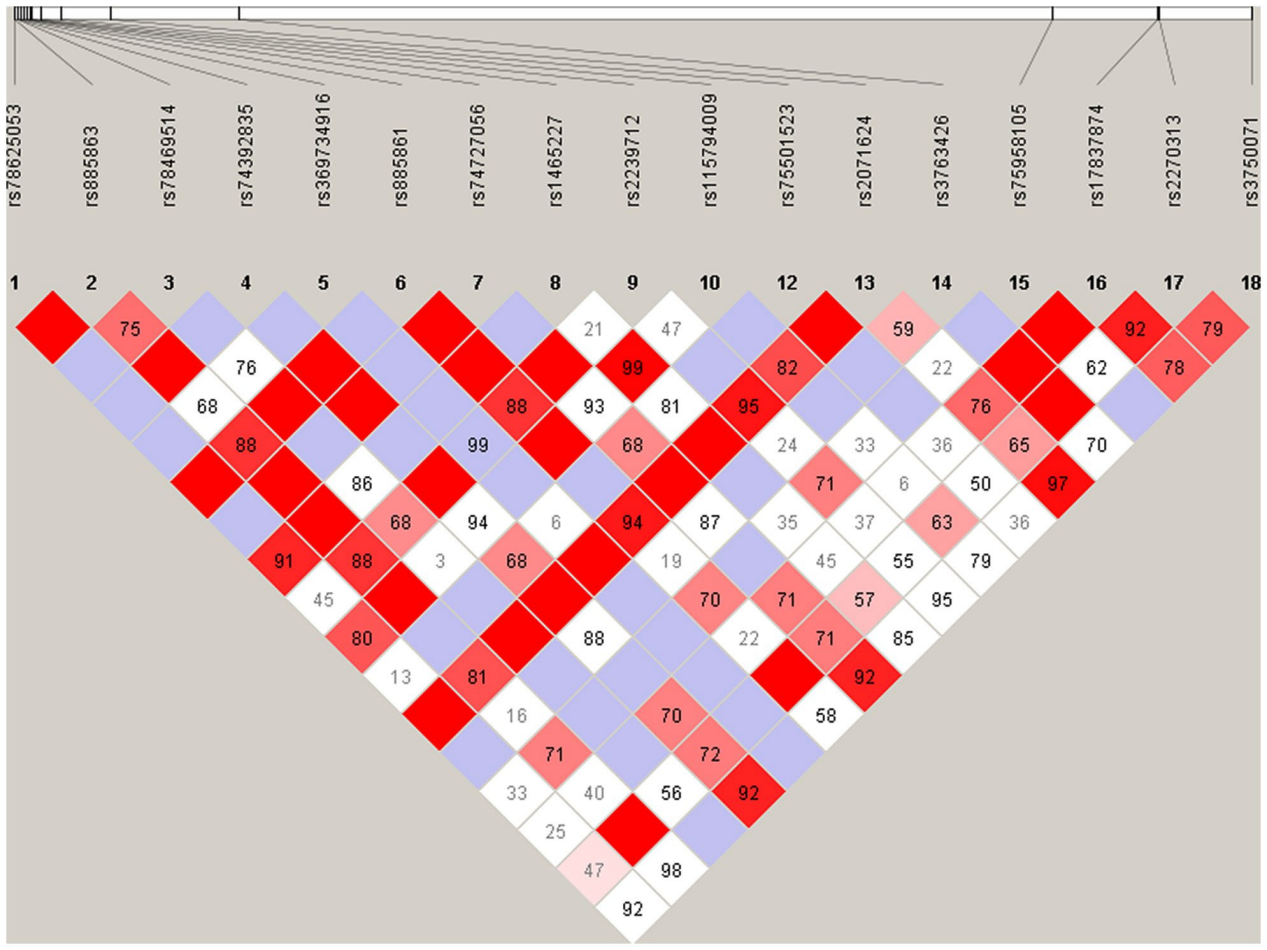
Pairwise linkage disequilibrium plot for the common variants in *VIPR2* gene. The pairwise *D*′ values are presented in the matrices, Deep red implicates relatively strong linkage disequilibrium, and vice versa.

Hardy–Weinberg equilibrium analysis was performed for all the loci, and seven loci, including two rare and five common variants, were eliminated from further study because their HWE *p*-values in control group were less than 0.05. The results of pairwise linkage disequilibrium analysis showed that there was no haplotype block identified (Figure 5).

After FDR-BH correction, three rare mutations, including one non-synonymous variant and two intron variants, achieved significant association with SCZ (Table 3). Two alter alleles of the non-synonymous variant, rs78564798 [NM_003382: c.709C > G/T, p.(Pro237Ala)/p.(Pro237Ser)], were identified in two different cases while the alter allele “C” was carried by ten control samples (*P_allele_* = 0.006, *P_genotype_* = 0.006 after FDR-BH correction). One of the significant intron variants (chr7:159034078, GRCh38) was novel reported and the variant only detected in 14 cases suggesting that the alter allele “C” in this locus may be a risk factor of SCZ (*P_allele_* = 0.048, *P_genotype_* = 0.048 after FDR-BH correction). The other significant intron variant, rs372544903 (*P_allele_* = 0.026, *P_genotype_* = 0.028 after FDR-BH correction), was only found in the control group indicating that the alter allele “T” may be a protective factor of SCZ. Additionally, there was a nominally significant association between SCZ and the rare non-synonymous variant rs199630455 (*P_allele_* = 0.009, *P_genotype_* = 0.009, OR for “C” [95% CI] = 0.206 [0.054 ~ 0.778]), and the alter allele “C” may be a protective factor of SCZ, while the significance disappeared after correction.

**Table 3:** Association results of the variants.

Position	Group	Allele frequency		Allelic *P*	Corrected *P*^a^	OR	95% CI	Genotype frequency			Genotypic *P*	Corrected *P*^a^	Rare or common
chr7: 159034078		G	T					T/T	T/G				
Novel	SCZ	14(0.39%)	3,594(99.6%)	0.005**	0.048*	NA	NA	1790(99.2%)	14(0.78%)		0.005**	0.048*	Rare
Intronic	Control	0(0.00%)	1992(100%)					996(100%)	0(0%)				
chr7:159036791		A/C	G					G/G	G/A	C/G			
rs78564798	SCZ	1(0.03%) /1(0.03%)	3,606(99.9%)	2.38e-04**	0.006**	NA	NA	1802(99.9%)	1(0.05%)	1(0.05%)	4.76e-04**	0.006**	Rare
Non-synonymous SNV	Control	0(0%)/10(0.50%)	1982(99.5%)					986(99.0%)	0(0%)	10(1.00%)			
chr7:159103734		T	C					C/C	C/T				
rs372544903	SCZ	0(0%)	3,608(100%)	0.002**	0.026*	NA	NA	1804(100%)	0(0%)		0.002**	0.028*	Rare
Intronic	Control	5(0.25%)	1987(99.7%)					991(99.5%)	5(0.50%)				
chr7:159034586		T	C					T/T	C/T				
rs199630455	SCZ	3,605(99.9%)	3(0.08%)	0.021*	0.085	0.206	[0.054 ~ 0.778]	1801(99.8%)	3(0.17%)		0.021*	0.08	Rare
Non-synonymous SNV	Control	1984(99.6%)	8(0.40%)					988(99.2%)	8(0.80%)				

SCZ, schizophrenia; SNP, single nucleotide polymorphism; OR, odds ratio; CI, confidence interval. ^a^FDR correction. *P-values <0.05; **P-values <0.01.

We also carried out gene-based association studies for frameshift deletions and non-synonymous variations. In total, non-synonymous variants were detected in 54 patients with SCZ and 49 control samples. The cumulative numbers of non-synonymous mutation carriers in the case group and control group were substantially different according to the chi-square test (chi^2^ = 6.72, *p* = 0.009, Supplementary Table S4).

## Discussion

In this study, a total of 24 non-synonymous mutations and two frameshift deletions on the *VIPR2* gene were identified by amplicon targeted resequencing. Among them, six non-synonymous mutations and one frameshift deletion were detected only in the control group, as well as 11 non-synonymous mutations and one frameshift deletion were detected only in patients with SCZ. Only variants detected in the control group were predicted to be benign (tolerated or neutral) by at least two pathogenicity prediction tools suggesting that some variants on the *VIPR2* gene may have little effect on the expression, structure, and function of the VPAC2 receptor. Additionally, a gene-based association study found that the control group carried significantly more non-synonymous mutations and frameshift deletions than the case group, indicating that the presence of non-synonymous mutations on the *VIPR2* gene, the majority of which are predicted to be benign, may prevent the *VIPR2* gene’s overexpression or overactivation of its downstream pathways, offering protection against SCZ.

Four detrimental non-synonymous variants that predicted by at least three tools, namely, *VIPR2* (NP_003373): p.(Trp282Cys), p.(Arg423Ser), p.(Gly317Ser), and p.(Arg63Trp), were only detected in patients with SCZ. The fact that these variations are highly conserved across species raises the possibility that the amino acid changes at these locations may have a significant effect on the structure and functionality of the VPAC2 receptor and subsequently contribute to the molecular pathogenesis of SCZ. One of them, p.(Arg63Trp), is found in the extracellular domain of the protein, and this mutation may have an impact on how the receptor binds to its ligand. Mutations p.(Arg423Ser) and p.(Gly317Ser) in the protein’s intracellular domain may impact the activation of the downstream pathway, while p.(Trp282Cys) in the helical transmembrane domain may affect the protein’s location on the cell membrane. Additionally, the SCHEMA browser database^7^ also reported the variants p.(Val297Phe), p.(Trp282Cys), p.(Asp113Asn), and p.(Val106Ile), which were discovered in individuals with SCZ in our investigation. We conducted meta-analysis for these loci, but no significance was found. Recently, five missense SNVs in the VIPR2 gene were identified in 516 patients with SCZ (Chen et al., 2022). Only p.(Thr189Lys) was found in our sample’s control group, and none of the loci found in our cases were reported in their research. Our research adds to the body of information on exon sequencing of the *VIPR2* gene in the Chinese Han population and presents a comprehensive mutation spectrum of the *VIPR2* gene for SCZ.

Our study identified several variants in the *VIPR2* gene that may affect the expression and function of VPAC2, while the role of VPAC2 in postnatal maturation of the nervous system has been demonstrated, which is vital for the normal development of circuits crucial to cognition. VPAC2 and PAC1 are the receptors for PACAP and VIP. It has been confirmed *in vitro* using cultured neurons that VPAC2 activation by PACAP and VIP inhibits axons and dendrites growth while PAC1 receptor activation by PACAP stimulates growth (Ago et al., 2021). It has been reported that PACAP as a ligand for VPAC2, is significantly associated with SCZ and risperidone can reverse the aberrant behaviors shown in PACAP^−/−^ mice, which are thought to represent schizophrenia-like phenotypes in rodents (Hashimoto et al., 2007). Additionally, mice who were continuously treated with PCP and utilized as prospective animal models for SCZ had considerably less PACAP mRNA expressed in the frontal cortex (Hashimoto et al., 2007). On the other hand, *VIPR2* is involved in cAMP signaling and the activation of PKA, which in turn affects the hippocampus’s *N*-methyl-d-aspartate (NMDA) receptor-mediated synaptic transmission (Yang et al., 2009; Tam et al., 2015). VIP is another ligand for VPAC2, and Vip-deficient mice display a decline in hippocampus-dependent associative memory (Chaudhury et al., 2008), a cognitive impairment frequently observed in patients with SCZ (Libby et al., 2013; Tam et al., 2015).

Our results provided additional evidence that *VIPR2* was a susceptibility gene of SCZ. However, there are two limitations of our study. The first is the small sample size, and the second is the absence of functional validation. To further understand the etiology associated with the *VIPR2* gene in SCZ, targeted sequencing for the *VIPR2* gene in more patients with the disorder and more functional validations are thought to be necessary.

In conclusions, the comprehensive mutation spectrum of the *VIPR2* gene in SCZ was revealed by our research. In *VIPR2*, we discovered a significant difference between the overall number of rare non-synonymous mutation carriers in SCZ cases and healthy controls. Significant associations between SCZ and the non-synonymous mutation rs78564798 as well as two newly discovered rare variations in the *VIPR2* gene’s introns have been found. Six novel rare non-synonymous variants and two novel frameshift deletions were reported in our study. The amino acid sequences of the five loci that were found in the case group were highly conserved, and according to functional prediction, their mutations may influence the structure and function of VPAC2 and thus be involved in the pathogenesis of SCZ. All the results further supported the hypothesis that the *VIPR2* gene is a susceptibility gene for SCZ, but further functional validations are required to understand the role of *VIPR2* in the etiology of SCZ.

## Data availability statement

The original contributions presented in the study are included in the article/Supplementary material, further inquiries can be directed to the corresponding authors.

## Ethics statement

The studies involving human participants were reviewed and approved by Nanjing Medical University. The reference number is [2019]166. The patients/participants provided their written informed consent to participate in this study.

## Author contributions

JMY and ZZ designed and supervised the whole research process. JJY and JZ carried out all experiments and managed the literature searches. FF and SY conducted DNA extraction and Sanger sequencing. JJY and JW undertook the statistical analysis. JMY and JJY conducted the patient recruitment and diagnosis as well as sample collection. JZ wrote the first draft of the manuscript. JJY, JMY, and ZZ proof read the article. All authors contributed to and approved the final manuscript.

## Funding

This study was supported by Wuxi Taihu Talent Project (Nos. WXTTP2020008 and WXTTP2021), Wuxi Municipal Health Commission (Q202131), and Science and Technology Development Foundation of Nanjing Medical University (NMUB2020292), Medical Research Project of Jiangsu Province Health Commission (M2020078).

## Conflict of interest

The authors declare that the research was conducted in the absence of any commercial or financial relationships that could be construed as a potential conflict of interest.

## Publisher’s note

All claims expressed in this article are solely those of the authors and do not necessarily represent those of their affiliated organizations, or those of the publisher, the editors and the reviewers. Any product that may be evaluated in this article, or claim that may be made by its manufacturer, is not guaranteed or endorsed by the publisher.

## Footnotes

^1^ https://software.broadinstitute.org/gatk

^2^ http://www.ebi.ac.uk/Tools/msa/clustalo/

^3^ http://shesisplus.bio-x.cn/SHEsis.html

^4^ https://www.broadinstitute.org/haploview

^5^ http://genetics.bwh.harvard.edu/pph2

^6^ http://www.mutationtaster.org

^7^ http://schema.broadinstitute.org/results

## Abbreviations

VIPR2: vasoactive intestinal peptide receptor 2 gene
SCZ: schizophrenia
ADHD: attention deficit and hyperactivity disorder
MD: mood disorders
SNPs: single-nucleotide polymorphisms
CNVs: copy number variations
GPCR: G-protein-coupled receptor
VIP: vasoactive intestinal peptide
PACAP: pituitary adenylate cyclase-activating polypeptide
CNS: the central nervous system
AC: adenylate cyclase
PKA: protein kinase A
PLC: phospholipase C
SNVs: single nucleotide variants
UTR: un-translated region
GATK: Genome Analysis Toolkit
BWA: Burrows-Wheeler Aligner
InDels: short insertions and deletions
HWE: Hardy–Weinberg equilibrium
FDR: false discovery rate
LD: linkage disequilibrium
MAF: minor allele frequency
CADD: Combined Annotation Dependent Depletion
NMDA: *N*-methyl-d-aspartate
