# Supplementary figures and images for: Identification of VIPR2 rare and common variants in the Chinese Han population with schizophrenia
Source: Front Mol Neurosci. 2023 Apr 27;16:1170708. doi: 10.3389/fnmol.2023.1170708 (PMC10174236; doi:10.3389/fnmol.2023.1170708)

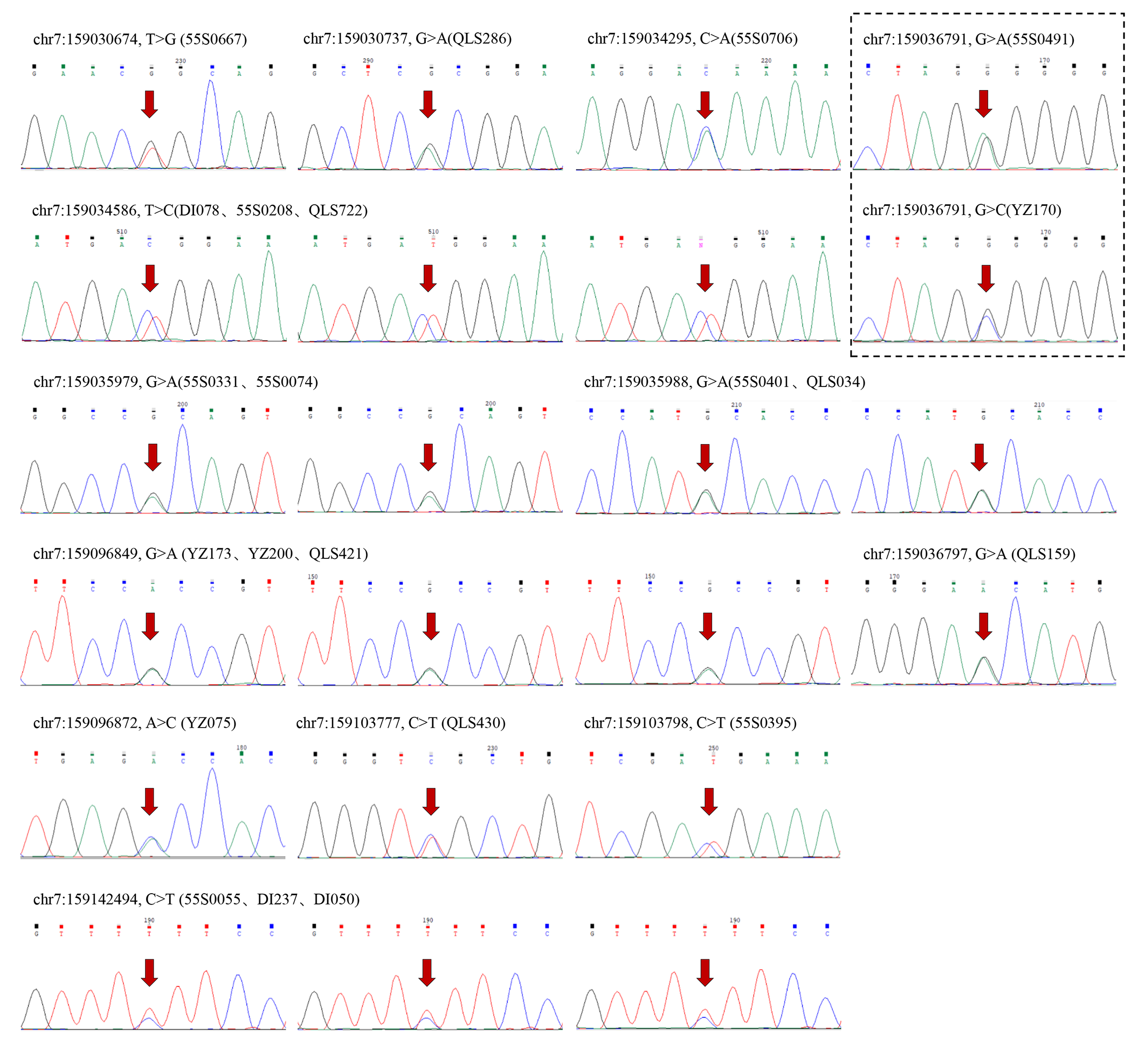

Supplement: Supplementary file 2 [file Image_1.TIF]
